# Association study in three different populations between the GPR88 gene and major psychoses

**DOI:** 10.1002/mgg3.54

**Published:** 2013-12-12

**Authors:** Maria Del Zompo, Jean-François Deleuze, Caterina Chillotti, Emmanuelle Cousin, Dana Niehaus, Richard P Ebstein, Raffaella Ardau, Sandrine Macé, Louise Warnich, Mustafa Mujahed, Giovanni Severino, Colette Dib, Esme Jordaan, Ibrahim Murad, Stéphane Soubigou, Liezl Koen, Issam Bannoura, Corinne Rocher, Claudine Laurent, Murielle Derock, Nicole Faucon Biguet, Jacques Mallet, Rolando Meloni

**Affiliations:** 1 Section of Neurosciences and Clinical Psychopharmacology, Department of Biomedical Sciences, University of CagliariCagliari, Italy; 2 Unit of Clinical Pharmacology, Teaching Hospital of Cagliari, AOUCACagliari, Italy; 3 Sanofi13 quai Jules Guesde, Vitry, France; 4 Department of Psychiatry, Faculty of Health Sciences, Stellenbosch UniversityStellenbosch, South Africa; 5 Psychology Department, National University of SingaporeSingapore; 6 Department of Genetics, Stellenbosch UniversityStellenbosch, South Africa; 7 Palestinian Research Center for Genetics of Mental Disorders, Bethlehem Mental HospitalBethlehem, Palestine; 8 Biostatistics Unit, Medical Research CouncilBellville, South Africa; 9 Department of Child and Adolescent Psychiatry, Pierre and Marie Curie Faculty of MedicineParis, France; 10 Department of Biotechnology and Biotherapy C.R.I.C.M. UPMC/INSERM UMR_S975/CNRS UMR 7225, Institut du Cerveau et de la Moelle épinière (ICM)GHU Pitié-Salpêtrière, Paris, France

**Keywords:** Candidate gene, homogeneous populations, Palestine, Sardinia, South Africa

## Abstract

*GPR88*, coding for a G protein-coupled orphan receptor that is highly represented in the striatum, is a strong functional candidate gene for neuropsychiatric disorders and is located at 1p22-p21, a chromosomal region that we have previously linked to bipolar disorder (BD) in the Sardinian population. In order to ascertain the relevance of *GPR88* as a risk factor for psychiatric diseases, we performed a genetic association analysis between *GPR88* and BD in a sample of triads (patient and both parents) recruited in the Sardinian and the Palestinian population as well as between *GPR88* and schizophrenia (SZ) in triads from the Xhosa population in South Africa. We found a positive association between *GPR88* and BD in the Sardinian and Palestinian triads. Moreover, we found a positive association between *GPR88* and SZ in triads from the Xhosa population in South Africa. When these results were corrected for multiple testing, the association between *GPR88* and BD was maintained in the Palestinian population. Thus, these results suggest that *GPR88* deserves consideration as a candidate gene for psychiatric diseases and requires to be further investigated in other populations.

## Introduction

A genome-wide scan on a sample of 88 nuclear families with at least two affected sibs with bipolar disorder (BD) recruited in the Sardinian population, showed positive nonparametric linkage (NPL) scores exceeding 3.4 with *P*-values <0.0003 with two adjacent markers, D1S206 and D1S435 at 1p22-p21, a chromosomal region that had never been linked to BD before. Moreover, a subsequent fine mapping of the 1p22-p21 region confirmed the suggestive linkage finding for this region with NPL values above 2.2 and *P*-values < 0.02 for a cluster of nine markers comprising D1S435 (Del Zompo et al. 2010).

The 1p21 chromosomal region contains the *GPR88* gene, encoding a G protein-coupled orphan receptor that is highly represented in the striatum (Mizushima et al. 2000). *GPR88* represents, among the genes located between the D1S206 and D1S435 markers, the most compelling a priori candidate gene as risk factors for psychiatric diseases. For instance, *Gpr88* expression is restricted to the projecting medium spiny neurons and is regulated by dopaminergic and glutamatergic afferents (Massart et al. 2009) as well as by methamphetamine, the mood stabilizers valproate (Ogden et al. 2004) and lithium (Brandish et al. 2005) and by antidepressant treatments (Conti et al. 2006). Moreover, the *Gpr88* knock down (KO) mice exhibit behavioral and psychotropic drug-associated responses (Logue et al. 2009) as well as cognitive impairments (Quintana et al. 2012) that reproduce several deficits characterizing neuropsychiatric diseases. Taken together, these findings suggest that *GPR88* may be implicated in the etiology of psychiatric diseases and has been heralded as potential therapeutic target both for BD (Ogden et al. 2004; Brandish et al. 2005) and schizophrenia (SZ) (Logue et al. 2009).

On the basis of the hypothesis that BD and SZ share common genetic causes (Lichtenstein et al. 2009; Williams et al. 2011), here we report the results of a genetic association study of *GPR88* for BD and SZ. We have conducted this association study using nine DNA markers covering the sequence of *GPR88* in nuclear families formed by a proband with BD and both parents (triads) originating from the Sardinian population and from the Palestinian population, as well as in a sample of SZ triads recruited in the Xhosa population from South Africa.

Significant associations were found at different markers between *GPR88* and BD in the Sardinian and Palestinian populations, as well as between *GPR88* and SZ in the Xhosa population. After correction for multiple testing, the association between *GPR88* and BD was still significant in the Palestinian population.

These results, stemming from a previous linkage analysis study, suggest that *GPR88* is not only a functional but also a positional candidate gene and, thus, requires further genetic studies in order to ascertain the extent of the putative implication of *GPR88* in the etiology of psychiatric diseases.

## Material and Methods

### Family collection and diagnostic control

#### Sardinian sample

A sample of 108 nuclear families constituted by one affected child and the two parents (triads) was recruited over a 2-year period in Sardinia. These families were independent from and do not overlap with the sib-pair sample previously used for the genome-wide linkage analysis that some of us have performed in the Sardinian population (Del Zompo et al. 2010). Only patients of Sardinian descent for at least three generations and with living parents were included in the study. Lifetime consensus diagnoses according to DSM-IV criteria (A.P.A. 1994) were achieved by trained physicians using data from a personal structured interview (Schedule for Affective Disorder and Schizophrenia-Lifetime Version) (Endicott and Spitzer 1978), Operational Criteria System (OPCRIT) documentation (McGuffin et al. 1991) and a systematic review of the medical records. The probands, 52 men and 56 women, were diagnosed as Bipolar I disorder. The mean age of onset (± SD) was 21.71 ± 6.51 years and the age (± SD) at the moment of the interview was 32.66 ± 8.39 years. We used the Family History-Research Diagnostic Criteria (FH-RDC) to collect all available data on the family history of our probands (Andreasen et al. 1977). Approval for the study was obtained from the local research ethic committee. After a complete description of the study, informed consent was obtained from all subjects and blood samples were taken from probands and their parents.

#### Palestinian sample

A total of 82 triads, including 52 male and 30 female bipolar patients, were collected. All families were of Palestinian Arab ancestry (at least two generations) and recruited in the West Bank mainly in the Bethlehem and East Jerusalem areas. The patients were interviewed by an experienced psychiatrist using the Structured Clinical Interview for DSM-IV (SCID) interview. The diagnosis of BD was assigned on the basis of the interview and medical records according to DSM-IV criteria (A.P.A. 1994). Included probands were diagnosed as bipolar I disorder. All patients gave informed consent and the protocol was approved by the local Institutional Review Board committee.

#### South-African sample

A sample of 171 triads was recruited over a 4-year period in the Western, Southern, and Eastern Cape provinces of South Africa. Mental health workers were asked to identify possible participants with clinical features of schizophrenia, who were then screened for suitability. Informed, written consent, as approved by the ethics committee of the Faculty of Health Sciences of Stellenbosch University, was obtained from all subjects before including them in the study. Participants had to be of Xhosa ethnicity, defined as reporting four grandparents of Xhosa descent. The lifetime consensus diagnoses according to DSM-IV criteria (A.P.A. 1994), were based on the data derived from the Diagnostic Interview for Genetic Studies (DIGS) version 2.0 (Nurnberger et al. 1994), OPCRIT documentation (McGuffin et al. 1991) and a review of the medical records. A trained psychiatrist and a Xhosa-speaking psychiatric nurse with extensive clinical experience assessed each participant using an English version of the DIGS, which was verbally translated into Xhosa. Regular calibration meetings were held. The probands, 137 men and 34 women, were diagnosed with schizophrenia (SZ). The mean age of onset (± SD) was 22 (± 5.1) years and the age (± SD) at the moment of the interview was 33 (± 8.0) years.

### Marker selection and genotyping

A total of nine single nucleotide polymorphism (SNP) markers covering ∼3.4 kb of genomic DNA encompassing the *GPR88* gene sequence were selected from dbSNP database (http://www.ncbi.nlm.nih.gov/SNP/) as well as Incyte private database (http://www.incyte.com). Only SNPs with reported allele frequency data were selected. These SNPs were distributed as follows: one SNP in the 5′ region of the gene, two located in the 5′UTR region, one in the protein coding sequence, and five in the 3′UTR region (Table 1). The rs2809819 marker, located in the second exon, is a non synonymous SNP leading to an Ile->Val variation at position 190 in the protein sequence.

**Table 1:** List of selected markers for the GPR88 gene.

#	Marker/rsID	Polymorphism	AA variation	Position
1	rs2036212	G/A		5′gene
2	rs2809823	A/C		5′UTR
3	rs2809822	T/C		5′UTR
4	rs2809819	A/G	I190V	Exon 2
5	rs2809818	C/T		3′UTR
6	iSNP00034643	C/T		3′UTR
7	rs2030048	A/G		3′UTR
8	rs2809817	T/C		3′UTR
9	rs2030049	T/C		3′UTR

The genotypes were determined after polymerase chain reaction and 5′ nuclease assay chemistry protocol (allelic discrimination with ABI TaqMan-specific probes) and read on an ABI 7900HT (Life Technologies SAS, Division Applied Biosystems, Villebon sur Yvette, France).

The polymerase chain reaction (PCR) primers and probes were chosen according to the supplier to amplify the nine loci (Table S1). The genotypes were determined using the SDS2.0 software. All reagents and software used are licensed to Applied Biosystems.

At least two technicians independently checked the genotyping profiles; discrepancies were resolved and unreliable data removed.

### Statistical analysis

Linkage disequilibrium (LD) was estimated in each population (Sardinian/Palestinian/Xhosa) using the genotypes from the unrelated parents. Delta square (Δ^2^), the square of the standardized LD measure, was calculated according to published method (Devlin and Risch 1995).

The association with BD or SZ was individually tested for each marker using the transmission disequilibrium test (TDT) (Spielman et al. 1993). This test contrasts the alleles transmitted and not transmitted from parents heterozygous at the marker locus. The equality of these two proportions is tested using a binomial (asymptotically χ²) test with one degree of freedom. The TDT was performed using the TDTVAR program (Dudbridge et al. 2000). When the total number of transmissions (number of alleles transmitted and not transmitted) considered for the TDT was <25, an exact test was computed to reestimate the *P*-value. The False Discovery Rate (FDR) procedure (Benjamini and Hochberg 1995) was applied to adjust the results for multiple testing.

Multiple marker haplotype transmission was also investigated using version 2.5.4 of the TRANSMIT software (Clayton and Jones 1999). Two statistics were calculated: a chi-square with one degree of freedom (df) to test for a transmission excess of each specific haplotype and a global chi-square test to assess the overall transmission distortion for all the haplotypes. Haplotypes with a frequency <1% were not individually tested for transmission distortion. The global chi-square test was performed considering common haplotypes (i.e., haplotypes with frequency >1%) and a new haplotype created by pooling haplotypes with frequency <1%.

## Results

### Positioning of *GPR88* in the linkage region

The *GPR88* gene is located in the region previously found to be linked to BD in the Sardinian population (Del Zompo et al. 2010). More specifically, it is less than 4000 kb on the telomeric side of D1S293, which with D1S424 and D1S2804 constitutes a block of markers displaying NPL scores exceeding 2.35. Also, *GPR88* is less than 700 kb on the centromeric side of D1S206, which is part of a series of markers displaying NPL score above 1.7, according to the results obtained using estimated allele frequencies in our previous linkage study (Del Zompo et al. 2010).

### Linkage disequilibrium patterns

Linkage disequilibrium between paired SNP markers of the *GPR88* gene was estimated within each population using the genotypes of the parents (Table S2). In most of the cases, the amount of linkage disequilibrium between markers was small or null. Two pairs of markers, however, were in strong linkage disequilibrium (rs2809823/ rs2809819 and rs2809818/rs2030049) but this pattern was not found in the Xhosa population, probably because linkage disequilibrium is usually of shorter range in African populations.

### Association analysis

#### TDT analysis of individual markers

The association analysis conducted in the Sardinian triads resulted in a raw significant association, *P*-value = 0.037, between BD and the rs2030048 marker located in the 3′UTR of the *GPR88* gene. A raw significant association, *P*-value = 0.006, was also obtained in the similar sample of 82 BD patients and their respective parents recruited in the Palestinian population for the rs2809817 variant, a marker also located in the 3′UTR nearby to the rs2030048 marker (Table 2). When the two datasets were pooled, the associations were not maintained, as the pattern of transmission of the minor allele at each of the markers displaying association with BD was not consistent in the two populations (Table 2).

**Table 2:** Bipolar disorder TDT analysis results.

#	Marker/rsID	Population(s)														
		Sardinian					Palestinian					Total (Sardinian + Palestinian)				
		N_T_^1^	N_NT_^1^	TDT *P*-value	OR	95% C.I.	N_T_^1^	N_NT_^1^	TDT *P*-value	OR	95% C.I.	N_T_^1^	N_NT_^1^	TDT *P*-value	OR	95% C.I.
1	rs2036212	1	0	0.500^2^	n.a^3^	n.a^3^	6	10	0.454^2^	0.60	[0.22–1.65]	7	10	0.629^2^	0.70	[0.27–1.84]
2	rs2809823	55	46	0.370	1.20	[0.81–1.77]	34	45	0.216	0.76	[0.48–1.18]	89	91	0.882	0.98	[0.73–1.31]
3	rs2809822	–	–	n.d.^4^	n.d.^4^	n.d.^4^	–	–	n.d.^4^	n.d.^4^	n.d.^4^	–	–	n.d.^4^	n.d.^4^	n.d.^4^
4	rs2809819	60	48	0.248	1.25	[0.86–1.83]	33	43	0.251	0.77	[0.49–1.21]	93	91	0.883	1.02	[0.77–1.36]
5	rs2809818	15	18	0.602	0.83	[0.42–1.65]	14	20	0.303	0.70	[0.35–1.39]	29	38	0.272	0.76	[0.47–1.24]
6	iSNP00034643	42	31	0.198	1.35	[0.85–2.15]	22	23	0.882	0.96	[0.53–1.72]	64	54	0.357	1.19	[0.83–1.70]
7	rs2030048	**40**	**61**	**0.037**	**0.66**	**[0.44**–**0.98]**	36	29	0.385	1.24	[0.76–2.02]	76	90	0.277	0.84	[0.62–1.15]
8	rs2809817	24	35	0.152	0.69	[0.41–1.15]	**37**	**17**	**0.006**	**2.18**	**[1.23**–**3.87]**	61	52	0.397	1.17	[0.81–1.70]
9	rs2030049	15	16	0.857	0.94	[0.46–1.90]	15	20	0.398	0.75	[0.38–1.46]	30	36	0.460	0.83	[0.51–1.35]

Values in bold represent significant association.

1 Number of minor alleles transmitted (N_T_)/non transmitted (N_NT_).

2 *P*-value obtained by exact test.

3 Not applicable.

4 Not done because no allele variation was observed in this population.

The study of SZ triads from the Xhosa, a Bantu-speaking South-African population, yielded a raw significant association between SZ and two markers: rs2809819, *P*-value = 0.027, and iSNP00034643, *P*-value = 0.034 (Table 3), located, respectively, in the coding and 3′UTR regions (Table 1). The rs2809819 polymorphism corresponds to an I190V amino acid substitution that should not affect the function of the protein, as isoleucine and valine are both amino acids with aliphatic side chain yielding to homologous substitutions.

**Table 3:** Schizophrenia TDT analysis results.

#	Marker/rsID	Population				
		Xhosa				
		N_T_^1^	N_NT_^1^	TDT *P*-value	OR	95% C.I.
1	rs2036212	45	51	0.540	0.88	[0.59–1.32]
2	rs2809823	39	29	0.225	1.34	[0.83–2.17]
3	rs2809822	11	20	0.106	0.55	[0.26–1.15]
4	rs2809819	**34**	**18**	**0.027**	1.89	[1.07–3.34]
5	rs2809818	78	67	0.361	1.16	[0.84–1.61]
6	iSNP00034643	**22**	**10**	**0.034**	2.20	[1.04–4.65]
7	rs2030048	16	14	0.715	1.14	[0.56–2.34]
8	rs2809817	1	2	1.000^2^	0.50	[0.05–5.51]
9	rs2030049	72	86	0.265	0.84	[0.61–1.15]

Values in bold represent significant association.

1 Number of minor alleles transmitted (N_T_)/non transmitted (N_NT_).

2 *P*-value obtained by exact test.

After correction for multiple testing using the FDR procedure (Benjamini and Hochberg 1995), the rs2809817 marker still held a significant association (*P*-value = 0.048) with BD in the Palestinian population (Table 4).

**Table 4:** TDT analysis results adjusted for multiple testing.

#	Marker/rsID	Population (trait)					
		Sardinian (BD)		Palestinian (BD)		Xhosa (SZ)	
		Raw *P*-value	FDR adjusted *P*-value^1^	Raw *P*-value	FDR adjusted *P*-value^1^	Raw *P*-value	FDR adjusted *P*-value^1^
1	rs2036212	0.500^2^	0.667	0.454^2^	0.519	0.540	0.694
2	rs2809823	0.370	0.592	0.216	0.519	0.225	0.477
3	rs2809822	n.d.^3^	–	n.d.^3^	–	0.106	0.318
4	rs2809819	0.248	0.496	0.251	0.519	**0.027**	0.153
5	rs2809818	0.602	0.688	0.303	0.519	0.361	0.542
6	iSNP00034643	0.198	0.496	0.882	0.882	**0.034**	0.153
7	rs2030048	**0.037**	0.296	0.385	0.519	0.715	0.804
8	rs2809817	0.152	0.496	**0.006**	**0.048**	1.000^2^	1.000
9	rs2030049	0.857	0.857	0.398	0.519	0.265	0.477

Values in bold represent significant association.

1 *P*-value adjusted for multiple testing using the False Discovery Rate (FDR) procedure described by Benjamini and Hochberg (1995).

2 *P*-value obtained by exact test.

3 Not done because no allele variation was observed in this population.

#### Haplotype analysis

The transmission of haplotypes from parents to offspring was analyzed using TRANSMIT. This program is able to deal with transmission of multi-locus haplotypes even if parental genotypes are missing and if phase is unknown. The significance of transmission distortion was assessed by bootstrap (5000 bootstrap samples used for calculation). One haplotype showed a slightly significant excess transmission (*P *=* *0.021) in Xhosa SZ population but this finding was not observed in Sardinian and Palestinian BD populations (*P *=* *0.441 and *P *=* *0.690, respectively). Global association tests, that is, testing significance of transmission distortion for all the haplotypes, were not significant (Table S3).

## Discussion

Following the finding of positive linkage for BD in the p22-p21 region of chromosome 1 in the Sardinian population (Del Zompo et al. 2010), we have assessed the genetic association of *GPR88,* a candidate gene located in this region, both with BD and SZ.

The genetic component of BD and SZ is characterized by complex patterns of inheritance due to heterogeneity, polygenicity, and the interaction with the environment (Gejman et al. 2011). In order to attenuate the impact of these confounding factors, we have focused the association analysis on nuclear (triads) families recruited in the genetically homogeneous Sardinian, where we reported our initial positive linkage for BD on 1p22-p21 in a separate set of sib-pair families, Palestinian and South-African Xhosa populations. These populations represent geographic and/or historical genetic isolates that are characterized by lesser genetic heterogeneity and probably fewer environmental differences, and, thus, display a better signal-to-noise ratio in genetic studies than outbred populations (Lander and Kruglyak 1995; Escamilla 2001; Varilo and Peltonen 2004).

In this perspective, the Sardinian population, which is one of the best characterized genetic isolates in Europe (Cavalli-Sforza and Piazza 1996), is endowed, along with the Basque, Icelandic, and Finnish populations, with most of the ideal conditions, such as size, demographic stability, good genealogical records, and a modern health care system, that are instrumental for researching and identifying the genetic basis of complex diseases (Peltonen et al. 2000). The Palestinian population, albeit sharing several genetic characteristics with the Jewish populations in the region, constitutes also a genetic isolate descendent from a core settlement that may have lived in the area since prehistorical times (Nebel et al. 2000). Finally, the Xhosa population, belonging to the Bantu-speaking group, descends from an indigenous group who migrated in the early 1600s from southern Zaire to the Eastern Cape region of present South Africa and has remained relatively isolated since then (Seedat et al. 2004; Wright et al. 2010).

In addition to focusing on homogeneous populations, we have collected data for association studies on nuclear families composed of the patient with both parents in order to exploit the untransmitted alleles from the latter as internal controls by applying the TDT method. This approach avoids the risk of population stratification that, along with genetic heterogeneity, may hamper the validity of some genetic association studies. Moreover, the TDT is well suited for ascertaining association in presence of linkage (Ewens and Spielman 2005). Thus, we have conducted an association study in three different populations with homogeneous genetic structures and without recent significant admixture or gene flow and used a standardized methodological approach for the recruitment and the genetic analysis.

The association analysis revealed a genetic association between BD and a *GPR88* marker, rs2030048, in the Sardinian population. Interestingly, a positive association signal was also found with another marker, rs2809817, in a similar BD sample of Palestinian families. Then, we have combined the genotyping data obtained in these two populations in order to evaluate within a single sample the association of the gene with BD. Notwithstanding the increase in the sample size allowed by this approach, the combined analysis did not provide any positive result. However, this could be attributable to the genetic heterogeneity between populations. Finally, the extension of the association study of GPR88 to SZ revealed a positive association of two other GPR88 variants with SZ in the Xhosa population. The association with BD obtained with the marker rs2809817 in the Palestinian population was still significant after adjustment for multiple testing.

These findings may result by random spurious association as different alleles are implicated in the association in each population. However, the associations found may also reflect the different genetic landscapes and histories of these populations and support the notion of potential genetic heterogeneity (i.e., the markers implicated in the association being in linkage disequilibrium with different combinations of functional genetic variations having distinct effects on the risk for BD and SZ). Also, it is noteworthy that the association of SZ with the rs2809819 polymorphism, corresponding to an I190V amino acid substitution that should not affect the function of the protein, may still have a subtle and specific impact on some aspect of the genetic risk for SZ. This impact needs to be clarified by functional studies on these variants of *GPR88*.

A large amount of studies implicate a priori *Gpr88* in the pathophysiology of psychiatric diseases. For instance, the *Gpr88* gene encodes a G protein-coupled orphan receptor highly enriched in the striatum (Mizushima et al. 2000; Ghate et al. 2007; Van Waes et al. 2011) and whose expression, restricted to the projecting medium spiny neurons, is regulated by dopaminergic and glutamatergic afferents (Massart et al. 2009), two systems that have been implicated in the pathogenesis of SZ (Grace 1991; Carlsson et al. 2001) and BD (Cousins et al. 2009; Machado-Vieira et al. 2012). Moreover, transcriptomic studies have shown that *Gpr88* expression is regulated by lithium (Brandish et al. 2005), antidepressant (Conti et al. 2006) and psychostimulant treatments (Ogden et al. 2004).

The constitutive inactivation of *Gpr88* results in increased motor activity and disrupted prepulse inhibition (PPI), (Logue et al. 2009), which is a deficit in sensory-motor gating present in schizophrenic patients (Braff et al. 2001). Finally, the treatment with antipsychotic drugs (Logue et al. 2009) or the conditional re-expression of *Gpr88* in the striatum (Quintana et al. 2012) prevents behavioral and learning deficit associated with *Gpr88* KO.

Thus, our findings, taken together with functional data cumulated in the literature, suggest that further studies in other independent samples are needed in order to evaluate the relevance of *GPR88* in the genetic risk for BD and SZ. The development of these genetic studies along with the functional identification of the signaling pathways and gene and/or protein networks in which *GPR88* is implicated may offer new perspectives for the understanding of the etiology of psychiatric diseases and the development of new therapeutic approaches.

## Supporting Information

Table S1 PCR primers and probes.

Table S2 Linkage disequilibrium between paired SNP markers.

Table S3 Haplotype analyses.
