# Supplementary material for: Association study in three different populations between the GPR88 gene and major psychoses
Source: Mol Genet Genomic Med. 2013 Dec 12;2(2):152–9. doi: 10.1002/mgg3.54 (PMC3960057; doi:10.1002/mgg3.54)
Supplement: Table S1 — PCR primers and probes. [file mgg30002-0152-sd1.docx]

**Table S1**

**PCR primers and probes**

**Assay on Demand (Applied Biosystem design)**

| Marker / rsID | Marker Name | Assay ID | Context Sequence |  |
| --- | --- | --- | --- | --- |
| rs2036212 | GPR88u1_hCV11975892 | C__11975892_10 | CGTGGAAAGGCTGTCTTTTCCCTGG[A/G]TGATGTATAACTAAGCAGGAGAGAG |  |
| rs2809823 | GPR88e1_hCV2718868 | C___2718868_10 | GCTCTCCTCTTGAGCTCAGCTTCTG[A/C]TTTTGCAGCCAAGCATTCTTGCTGC |  |
| rs2809822 | GPR88e1_hCV26196627 | C__26196627_10 | ATGCTGTTGCTGAGGAGGTATTTCC[C/T]GGCATCCCTCCCCCTGAGACACCGG |  |

**Assay by Design**

| Marker / rsID | Marker Name | Forward Primer Sequence | Reverse Primer Sequence | Probe Reporter Sequence  (Dye VIC) | Probe Reporter Sequence  (Dye FAM) |
| --- | --- | --- | --- | --- | --- |
| rs2809819 | GPR88e2_hCV26196631 | CCTGGGCCTCGTGCT | GCAGCAGCAGAGCTGTCT | CACCGCGAATCCACTA | ACCGCGAGTCCACTA |
| rs2809818 | GPR88e2_hCV26196633 | GCGGGCTACCTGAACCAA | GTGGCCGTCCAAAATGACTTC | CCCCACTTAGAGAGACG | CCCACTTAAAGAGACG |
| iSNP00034643 | GPR88e2_iSNP00034643 | GGCAGTGAAGCCCTAGGT | CCTCATTTGAGAAAGACTTTATTAAAAACCCTT | TTGAGGTCTTAATGGCAAC | TTGAGGTCTTAATGACAAC |
| rs2030048 | GPR88e2_hCV2718870 | GCCTTATGAATGTGATTGCAGCTTT | CATTGAGATATTTTAAATGTTAAAGAGAAAACAGAAGGA | AACATTCTGTACTATAATGG | ACATTCTGTACTGTAATGG |
| rs2809817 | GPR88e2_hCV2718871 | CACACGTTAGAAAGCAACACTGTTT | TGTTCACTATATATGACTGAGTTTATTATACCTCCTT | TTGATGTTAAATGTCATGGTAAT | ATGTTAAATGTCGTGGTAAT |
| rs2030049 | GPR88e2_hCV11326901 | ACACGTTAGAAAGCAACACTGTTTTT | CCCATTTGAACTGTTCACTATATATGACTGA | TTCAACATATACAATATTG | CTTCAACATATACGATATTG |
