# Supplementary material for: Association study in three different populations between the GPR88 gene and major psychoses
Source: Mol Genet Genomic Med. 2013 Dec 12;2(2):152–9. doi: 10.1002/mgg3.54 (PMC3960057; doi:10.1002/mgg3.54)
Supplement: Table S2 — Linkage disequilibrium between paired SNP markers. [file mgg30002-0152-sd2.docx]

**Table S2**

**Linkage disequilibrium between paired SNP markers**

|  |  | **Δ^2^** | | |
| --- | --- | --- | --- | --- |
| **Marker A** | **Marker B** | **Sardinian (BD)** | **Palestinian (BD)** | **Xhosa (SZ)** |
| rs2036212 | rs2809823 | 0.00 | 0.06 | 0.04 |
| rs2036212 | rs2809822 | n.d.^(a)^ | n.d.^(a)^ | 0.21 |
| rs2036212 | rs2809819 | 0.00 | 0.06 | 0.03 |
| rs2036212 | rs2809818 | 0.02 | 0.35 | 0.13 |
| rs2036212 | iSNP00034643 | 0.00 | 0.00 | 0.01 |
| rs2036212 | rs2030048 | 0.00 | 0.03 | 0.01 |
| rs2036212 | rs2809817 | 0.00 | 0.01 | 0.00 |
| rs2036212 | rs2030049 | 0.02 | 0.36 | 0.29 |
| rs2809823 | rs2809822 | n.d.^(a)^ | n.d.^(a)^ | 0.01 |
| **rs2809823** | **rs2809819** | **0.94** | **0.92** | 0.53 |
| rs2809823 | rs2809818 | 0.12 | 0.16 | 0.11 |
| rs2809823 | iSNP00034643 | 0.19 | 0.12 | 0.40 |
| rs2809823 | rs2030048 | 0.42 | 0.45 | 0.18 |
| rs2809823 | rs2809817 | 0.16 | 0.25 | 0.03 |
| rs2809823 | rs2030049 | 0.12 | 0.16 | 0.03 |
| rs2809822 | rs2809819 | n.d.^(a)^ | n.d.^(a)^ | 0.00 |
| rs2809822 | rs2809818 | n.d.^(a)^ | n.d.^(a)^ | 0.03 |
| rs2809822 | iSNP00034643 | n.d.^(a)^ | n.d.^(a)^ | 0.00 |
| rs2809822 | rs2030048 | n.d.^(a)^ | n.d.^(a)^ | 0.00 |
| rs2809822 | rs2809817 | n.d.^(a)^ | n.d.^(a)^ | 0.00 |
| rs2809822 | rs2030049 | n.d.^(a)^ | n.d.^(a)^ | 0.06 |
| rs2809819 | rs2809818 | 0.13 | 0.17 | 0.19 |
| rs2809819 | iSNP00034643 | 0.18 | 0.14 | 0.56 |
| rs2809819 | rs2030048 | 0.42 | 0.44 | 0.29 |
| rs2809819 | rs2809817 | 0.16 | 0.20 | 0.04 |
| rs2809819 | rs2030049 | 0.13 | 0.17 | 0.09 |
| rs2809818 | iSNP00034643 | 0.03 | 0.03 | 0.11 |
| rs2809818 | rs2030048 | 0.05 | 0.07 | 0.10 |
| rs2809818 | rs2809817 | 0.02 | 0.04 | 0.01 |
| **rs2809818** | **rs2030049** | **0.94** | **0.94** | 0.41 |
| iSNP00034643 | rs2030048 | 0.14 | 0.13 | 0.00 |
| iSNP00034643 | rs2809817 | 0.05 | 0.06 | 0.00 |
| iSNP00034643 | rs2030049 | 0.03 | 0.03 | 0.05 |
| rs2030048 | rs2809817 | 0.38 | 0.48 | 0.09 |
| rs2030048 | rs2030049 | 0.05 | 0.07 | 0.04 |
| rs2030049 | rs2809817 | 0.02 | 0.04 | 0.00 |
| ^(a)^: not done because no allele variation was observed in this population | | | | |
