# Supplementary material for: Association study in three different populations between the GPR88 gene and major psychoses
Source: Mol Genet Genomic Med. 2013 Dec 12;2(2):152–9. doi: 10.1002/mgg3.54 (PMC3960057; doi:10.1002/mgg3.54)
Supplement: Table S3 — Haplotype analyses. [file mgg30002-0152-sd3.docx]

### Table S3

### Haplotype analyses

### Sardinian BD population

| **Haplotype^a^** | **Frequency** | Obs^b^ | **Exp^b^** | **Chi-square (1df)** | **P-value** |
| --- | --- | --- | --- | --- | --- |
| 1.2.1.2.1.1.1.1.1 | 0.338 | 79 | 72.99 | 1.54 | 0.284 |
| 1.1.1.1.1.2.1.1.1 | 0.197 | 46 | 42.46 | 0.67 | 0.441 |
| 1.1.1.1.1.1.2.1.1 | 0.181 | 34.93 | 39.19 | 1.10 | 0.357 |
| 1.1.1.1.1.1.2.2.1 | 0.165 | 30 | 35.74 | 2.22 | 0.243 |
| 1.2.1.2.2.1.1.1.2 | 0.090 | 18 | 19.50 | 0.27 | 0.624 |
| 1.2.1.1.1.1.1.1.1 | 0.012 | 3.06 | 2.59 | 0.18 | 0.657 |
| 1.1.1.2.1.1.1.1.1 | 0.005 | n.d.^c^ | | | |
| 1.2.1.2.1.2.1.1.1 | 0.005 | n.d.^c^ | | | |
| 1.1.1.1.1.1.1.1.1 | 0.002 | n.d.^c^ | | | |
| 1.2.1.2.1.1.1.1.2 | 0.002 | n.d.^c^ | | | |
| 2.2.1.2.1.1.1.1.2 | 0.002 | n.d.^c^ | | | |
| Global Chi-square test ^d^ (6 df): 5.68 Global p-value: 0.586 | | | | | |

| Palestinian BD population  \| **Haplotype^a^** \| **Frequency** \| Obs^b^ \| **Exp^b^** \| **Chi-square (1df)** \| **P-value** \| \| --- \| --- \| --- \| --- \| --- \| --- \| \| 1.2.1.2.1.1.1.1.1 \| 0.328 \| 49 \| 52.50 \| 0.71 \| 0.458 \| \| 1.1.1.1.1.1.2.2.1 \| 0.218 \| 44 \| 34.81 \| 6.57 \| 0.093 \| \| 1.1.1.1.1.2.1.1.1 \| 0.166 \| 25 \| 26.50 \| 0.20 \| 0.690 \| \| 1.1.1.1.1.1.2.1.1 \| 0.132 \| 18 \| 21.19 \| 1.19 \| 0.371 \| \| 1.2.1.2.2.1.1.1.2 \| 0.072 \| 10 \| 11.50 \| 0.53 \| 0.462 \| \| 2.2.1.2.2.1.1.1.2 \| 0.050 \| 6 \| 8.00 \| 1.00 \| 0.424 \| \| 1.2.1.2.1.2.1.1.1 \| 0.006 \| n.d.^c^ \| \| \| \| \| 1.2.1.1.1.1.2.1.1 \| 0.006 \| n.d.^c^ \| \| \| \| \| 1.1.1.2.1.1.2.2.1 \| 0.006 \| n.d.^c^ \| \| \| \| \| 1.2.1.1.1.1.1.1.1 \| 0.003 \| n.d.^c^ \| \| \| \| \| 1.2.1.1.1.2.1.1.1 \| 0.003 \| n.d.^c^ \| \| \| \| \| 1.1.1.1.2.1.2.2.1 \| 0.003 \| n.d.^c^ \| \| \| \| \| 1.2.1.2.1.1.1.1.2 \| 0.003 \| n.d.^c^ \| \| \| \| \| 1.2.1.2.2.1.2.1.2 \| 0.003 \| n.d.^c^ \| \| \| \| \| Global Chi-square test ^d^ (6 df): 9.68 \| \| \| \| \| \| \| Global p-value: 0.363 \| \| \| \| \| \| \| Xhosa SZ population  \| **Haplotype^a^** \| **Frequency** \| Obs^b^ \| **Exp^b^** \| **Chi-square (1df)** \| **P-value** \| \| --- \| --- \| --- \| --- \| --- \| --- \| \| 1.2.1.2.1.1.1.1.1 \| 0.235 \| 80.56 \| 80.31 \| 0.00 \| 0.963 \| \| 1.2.1.2.2.1.1.1.2 \| 0.226 \| 75.94 \| 77.15 \| 0.05 \| 0.825 \| \| 1.2.1.2.2.1.1.1.1 \| 0.195 \| 66.45 \| 66.66 \| 0.00 \| 0.965 \| \| 2.2.1.2.2.1.1.1.2 \| 0.151 \| 53 \| 51.48 \| 0.12 \| 0.719 \| \| 1.1.1.1.1.2.1.1.1 \| 0.054 \| 25 \| 18.53 \| 5.08 \| 0.021 \| \| 2.2.2.2.2.1.1.1.2 \| 0.048 \| 12 \| 16.52 \| 2.64 \| 0.058 \| \| 1.1.1.2.2.1.1.1.2 \| 0.034 \| 11 \| 11.54 \| 0.06 \| 0.765 \| \| 1.1.1.1.1.1.2.1.1 \| 0.023 \| 9.38 \| 7.70 \| 0.76 \| 0.377 \| \| 1.2.1.1.1.1.2.1.1 \| 0.010 \| 4 \| 3.51 \| 0.20 \| 0.726 \| \| 1.1.1.2.1.1.2.1.1 \| 0.009 \| n.d.^c^ \| \| \| \| \| 1.2.1.2.1.1.1.1.2 \| 0.008 \| n.d.^c^ \| \| \| \| \| 1.1.1.1.1.1.2.2.1 \| 0.004 \| n.d.^c^ \| \| \| \| \| 1.1.1.2.2.1.1.1.1 \| 0.001 \| n.d.^c^ \| \| \| \| \| 1.1.1.1.2.1.2.1.1 \| 0.001 \| n.d.^c^ \| \| \| \| \| 1.1.1.1.2.1.1.1.2 \| 0.001 \| n.d.^c^ \| \| \| \| \| 1.1.1.1.1.1.1.1.2 \| 0.001 \| n.d.^c^ \| \| \| \| \| Global Chi-square test ^d^ (9 df): 12.73 \| \| \| \| \| \| \| Global p-value: 0.146 \| \| \| \| \| \|   ^a^: Markers are shown in chromosomal order, as indicated in Table 1  ^b^: Obs/Exp are the observed/expected values used for chi-square calculation  ^c^: Not done / Haplotype with frequency <0.01, excluded from individual tests  ^d^: Global chi-square test performed from common haplotypes and the pool of haplotypes with frequency <1% \| \| \| \| \| \| |
| --- | --- | --- | --- | --- | --- | --- | --- | --- | --- | --- | --- | --- | --- | --- | --- | --- | --- | --- | --- | --- | --- | --- | --- | --- | --- | --- | --- | --- | --- | --- | --- | --- | --- | --- | --- | --- | --- | --- | --- | --- | --- | --- | --- | --- | --- | --- | --- | --- | --- | --- | --- | --- | --- | --- | --- | --- | --- | --- | --- | --- | --- | --- | --- | --- | --- | --- | --- | --- | --- | --- | --- | --- | --- | --- | --- | --- | --- | --- | --- | --- | --- | --- | --- | --- | --- | --- | --- | --- | --- | --- | --- | --- | --- | --- | --- | --- | --- | --- | --- | --- | --- | --- | --- | --- | --- | --- | --- | --- | --- | --- | --- | --- | --- | --- | --- | --- | --- | --- | --- | --- | --- | --- | --- | --- | --- | --- | --- | --- | --- | --- | --- | --- | --- | --- | --- | --- | --- | --- | --- | --- | --- | --- | --- | --- | --- | --- | --- | --- | --- | --- | --- | --- | --- | --- | --- | --- | --- | --- | --- | --- | --- | --- | --- | --- | --- | --- | --- | --- | --- | --- | --- | --- | --- | --- | --- | --- | --- | --- | --- | --- | --- | --- | --- | --- | --- | --- | --- | --- | --- | --- | --- | --- | --- | --- | --- | --- | --- | --- | --- | --- | --- | --- | --- | --- | --- | --- | --- | --- | --- | --- | --- | --- | --- | --- | --- | --- | --- | --- | --- | --- | --- | --- |
